# Treatment of Infected Tibia Non-union Using a Novel Solid Intramedullary Nail Custom-Made for Antibiotic-Impregnated Cement Coating

**DOI:** 10.7759/cureus.65918

**Published:** 2024-08-01

**Authors:** Ashwin Deshmukh, Abhishek Nair, Shivappa Devarmani, Ankit Barosani

**Affiliations:** 1 Orthopaedics, Dr. Dnyandeo Yashwantrao Patil Medical College, Hospital and Research Centre, Dr. Dnyandeo Yashwantrao Patil Vidyapeeth (Deemed to be University), Pune, IND

**Keywords:** intramedullary nail, antibiotics, cement, non-union, osteomyelitis

## Abstract

The chronic and incapacitating condition of infected non-union of the long bones continues to be a challenging issue for surgeons in terms of efficient and economical treatment. A number of variables, such as open fractures, soft tissue or bone loss, infection following internal fixation, persistent osteomyelitis with pathologic fractures, and surgical debridement of infected bone, can result in infected non-unions. An infected non-union is typically treated in two stages. To transform an infected non-union into an aseptic non-union, the initial step involves debridement, either with or without the insertion of antibiotic cement beads and systemic antibiotics. In order to ensure stability, external or internal fixation - with or without bone grafting - is carried out in the second stage. There is a wealth of literature supporting the use of antibiotic-impregnated cement-coated intramedullary (IM) nailing for infected non-union of tibia and femur fractures. In contrast to cement beads, the cement nail offers stability throughout the fracture site, and osseous stability is crucial for the treatment of an infected non-union. When using antibiotics for this purpose, they should possess unique qualities, including low allergenicity, heat stability, and a broad spectrum of activity. The most commonly utilised medication has been gentamicin, which is followed by vancomycin. Furthermore, it has been discovered that solid nails are more resistant to local infection than cannulated IM nails. In this case study, the patient was treated with a solid IM nail that had a specially designed slot on its exterior surface for the application of cement impregnated with antibiotics. In conclusion, an easy, affordable, and successful treatment for infected non-union of the tibia is antibiotic cement-impregnated nailing. It has strong patient compliance and removes the problems associated with external fixators, which makes it superior to them. A few benefits of this approach are early weight-bearing, stabilisation of the fracture, local antibiotic treatment, and the potential for accelerated rehabilitation. Additionally, lowering the requirement for continuous antibiotic medication may lessen the chance that antibiotic resistance may arise.

## Introduction

The chronic and incapacitating condition of infected non-union of the long bones continues to be a challenging issue for surgeons in terms of efficient and economical treatment [1]. A number of variables, such as open fractures, soft tissue or bone loss, infection following internal fixation, persistent osteomyelitis with pathologic fractures, and surgical debridement of infected bone, can result in infected non-unions [2]. The situation becomes more challenging when the implant that was used for internal fixation starts to grow biofilms and bacteria, making it a possible medium for infection [3].

An infected non-union is typically treated in two stages. To transform an infected non-union into an aseptic non-union, the initial step involves debridement, either with or without the insertion of antibiotic cement beads and systemic antibiotics. In order to ensure stability, external or internal fixation - with or without bone grafting - is carried out in the second stage [2].

There is a wealth of literature supporting the use of antibiotic-impregnated cement-coated intramedullary (IM) nailing for infected non-union of tibia and femur fractures. In contrast to cement beads, the cement nail offers stability throughout the fracture site, and osseous stability is crucial for the treatment of an infected non-union [4]. Antibiotic cement presents two advantages over systemic antibiotics: it permits a greater concentration of antibiotics at the local site and has fewer side effects involved. Refractory infection can be treated with antibiotic cement since it has been demonstrated to elute antibiotics at local locations for up to 36 weeks [4]. Therefore, in contrast to conventional methods of managing non-union infections, cement-coated nailing functions as a single-stage procedure, offering stability and treating infection simultaneously, along with additional benefits like early mobilisation, prevention of pin-site infections, ease of use, and cost-effectiveness.

When using antibiotics for this purpose, they should possess unique qualities, including low allergenicity, heat stability, and a broad spectrum of activity. The most commonly utilised medication has been gentamicin, which is followed by vancomycin [5]. The use of intramedullary nails coated with polymer or cement impregnated with antibiotics raises certain concerns, even with the encouraging results shown in the literature. The danger of antibiotic resistance is one of the main worries since the continued use of antibiotics can encourage bacterial strains that become resistant. The possibility that the cement or polymer coating could leak harmful compounds, which could cause tissue injury and slow healing, is another worry [6].

Furthermore, it has been discovered that solid nails are more resistant to local infection than cannulated intramedullary nails [7]. In this case study, the patient was treated with a solid intramedullary nail that has a specially designed facet on its exterior surface for the application of cement impregnated with antibiotics.

## Case presentation

A 48-year-old male came to our facility in January 2024 with complaints of discharge from his left leg and an inability to walk for three months. He had been a known case of type II diabetes mellitus and hypertension for one year and was on regular medications. He also had a history of tobacco chewing and consumption of alcohol for 10 years. He was apparently all right before September 2023, then he suffered a road traffic accident in September 2023, due to which he had a tibia and fibula mid-shaft fracture. He was operated on at an outside facility with closed reduction and internal fixation with intramedullary (IM) nailing for his left tibia shaft fracture, whereas the fibula shaft fracture was conserved. Two months post-surgery, he developed a discharging sinus over the distal part of his left leg. Suspecting infection, the intramedullary nail was removed, and external fixation was done for the non-united tibia in November 2023 at the same facility. Methicillin-resistant *Staphylococcus aureus* (MRSA) was detected in the intra-op sample, which was sent for culture sensitivity. Intravenous (IV) antibiotics were administered for a month, and the external fixator was removed in December 2023. The patient had no documentation of the antibiotic regimen or dosage.

**Figure 1:**
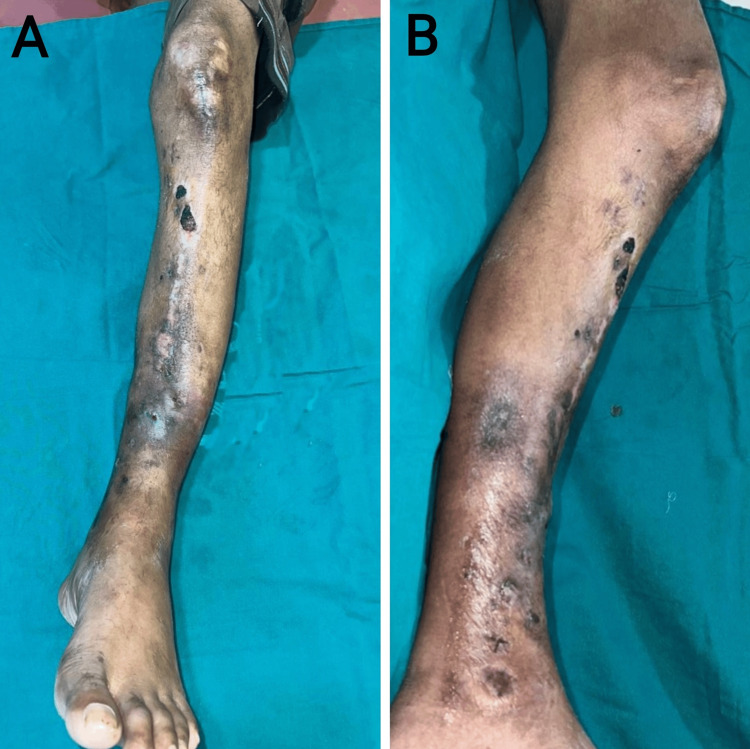
A) A clinical picture of the anterior aspect of the left leg. B) A clinical picture of the medial aspect of the left leg.

**Figure 2:**
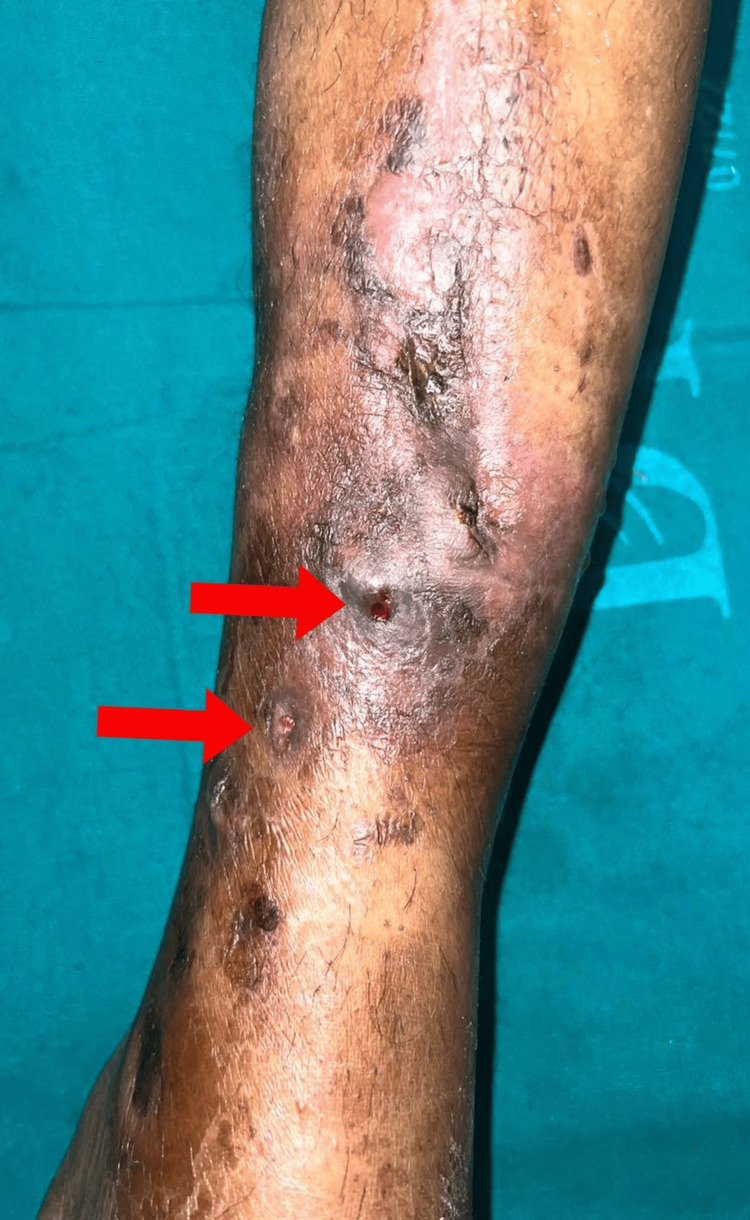
Red arrows show two discharging sinuses over the anteromedial aspect of the distal part of the left leg.

**Figure 3:**
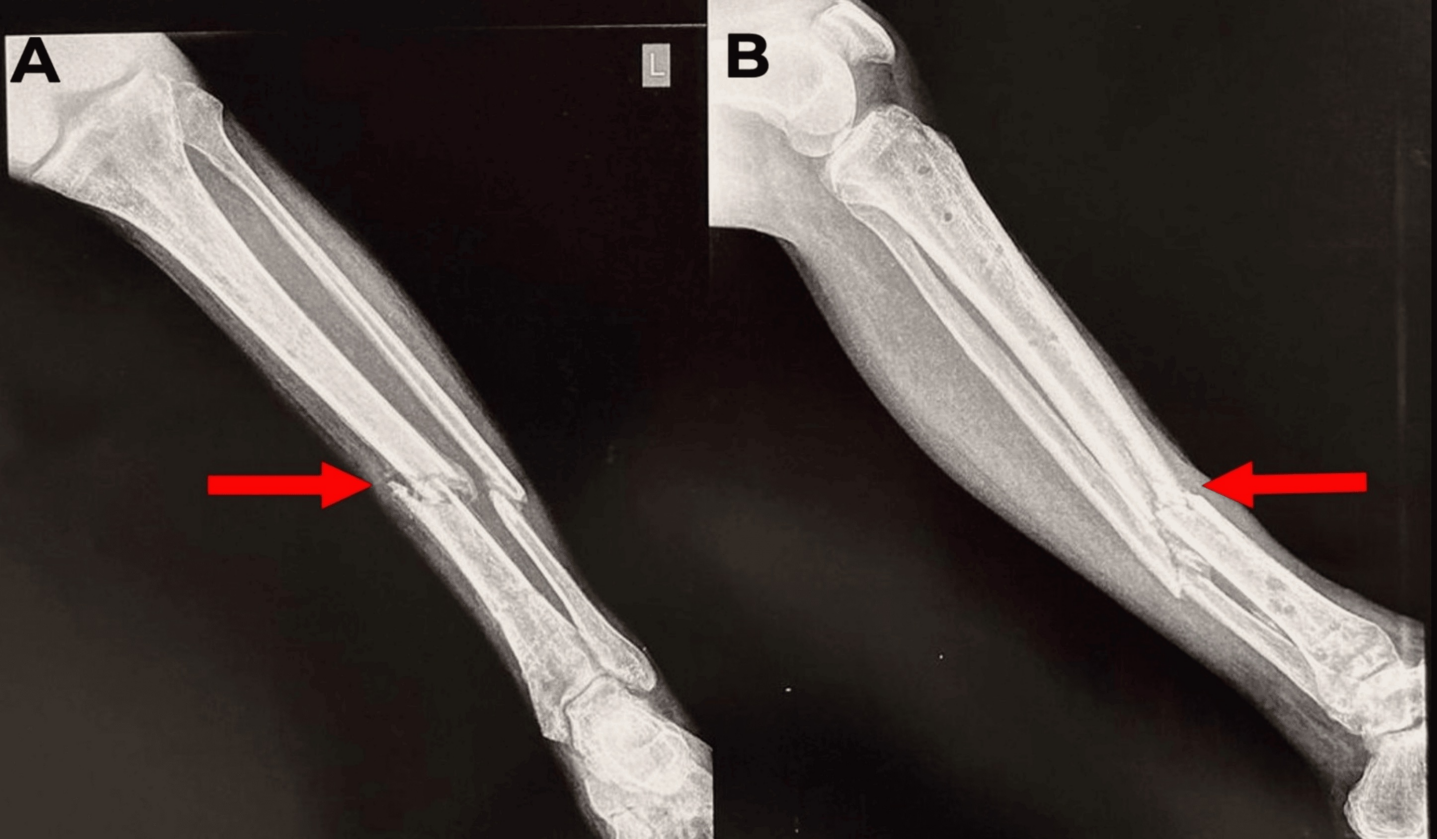
A) the left tibia and fibula X-ray AP view. B) left tibia and fibula X-ray lateral view. Red arrows show non-union of the tibia and fibula mid-shaft fracture. AP: anterior posterior

The patient then came to our facility in January 2024 with complaints of discharge from his left leg and an inability to walk since the removal of the external fixator. On examination, two 0.5 cm x 0.5 cm discharging sinuses were seen over the anteromedial aspect of the left distal tibia region and previous surgical scar marks (Figures 1-2). The sinuses were active with a scanty, seropurulent, yellowish, and non-foul-smelling discharge. The knee flexion range of motion was from 30-130 degrees, and there was no mobility at the ankle joint. There were no transmitting movements from the ankle joint to the knee joint. There was a visible bony deformity on the left leg. A full-length tibia-fibula anterior-posterior (AP) and lateral X-ray were done (Figure 3). The X-ray showed signs of tibia and fibula non-union at the mid-diaphysis. The discharge was collected on a swab stick and sent for culture sensitivity. The report showed MRSA positive, which was susceptible to vancomycin and gentamicin. This and the previous report from the outside facility showed a poor minimum inhibitory concentration (MIC) for gentamicin and vancomycin is considered as gold standard for MRSA; hence, we decided to use vancomycin for antibiotic-impregnated calcium sulphate beads and cement preparation, as well as IV administration, after discussing this with the infectious disease consultant and the microbiologist.

**Figure 4:**
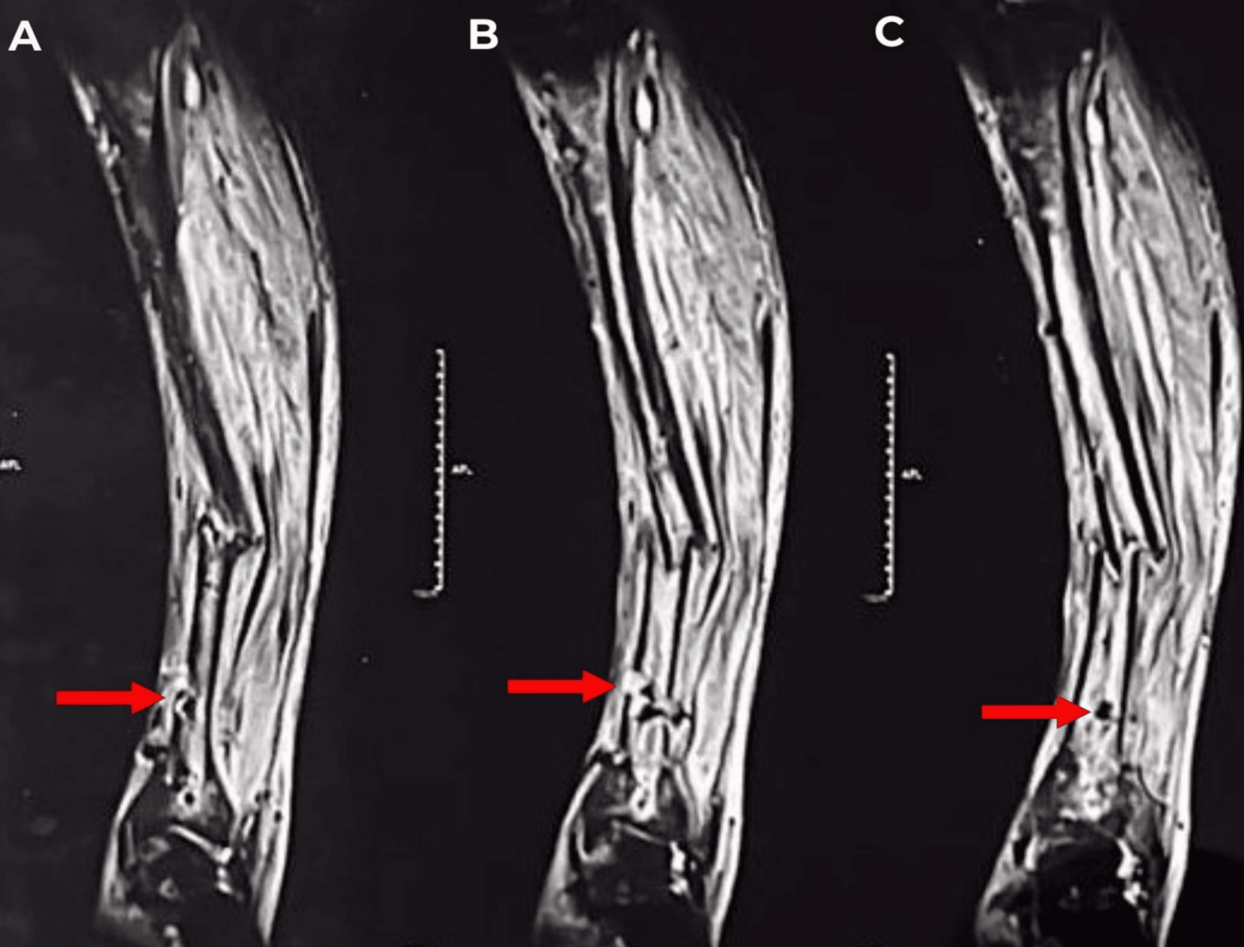
A-C) The sagittal section of the left leg MRI. Red arrows show a cortical breach in the lower one-third part of the tibia, through which a collection of fluid was seen. MRI: magnetic resonance imaging

**Figure 5:**
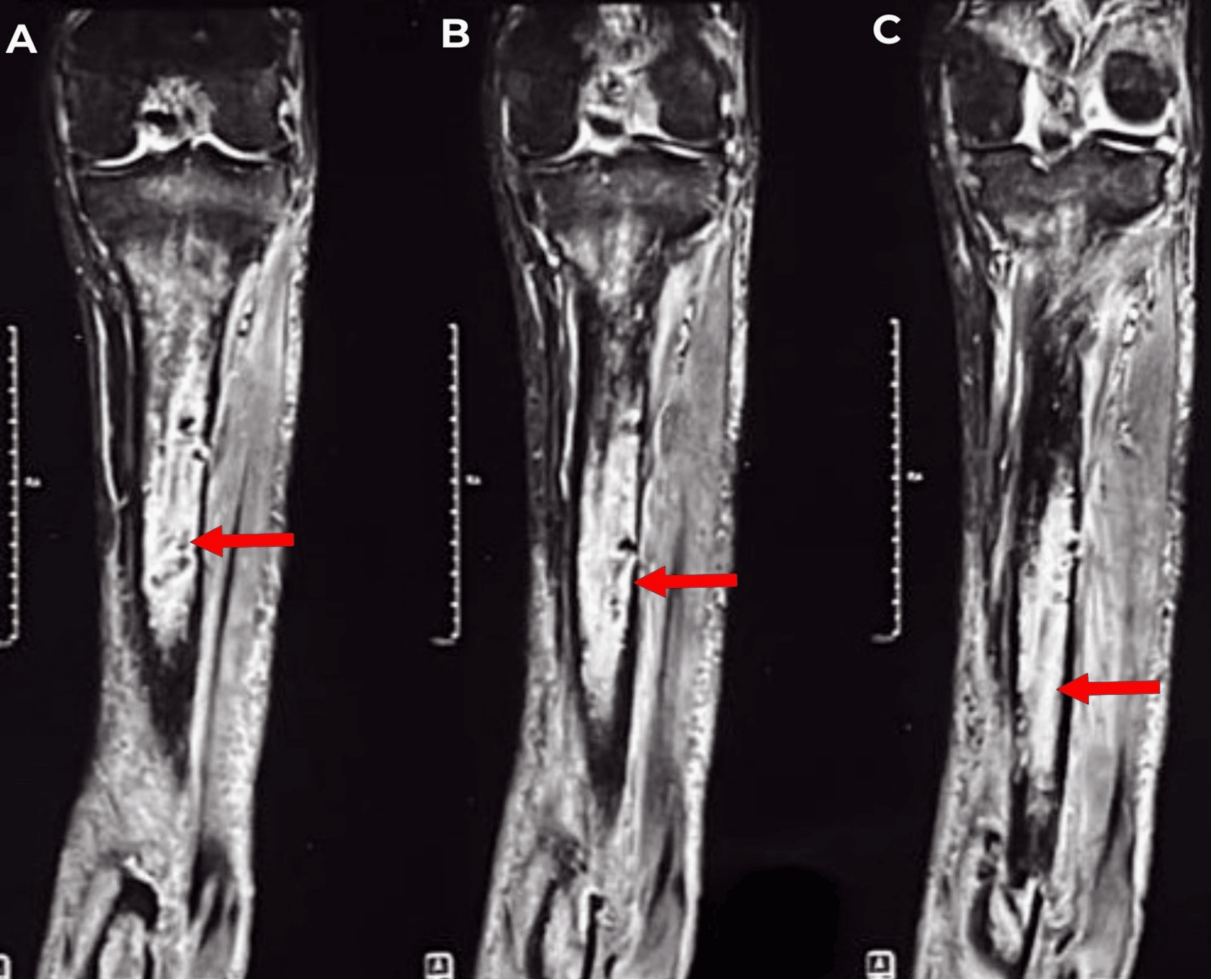
A-C) Coronal sections of the left leg MRI. Red arrows show fluid collection in the medullary canal of the tibia. MRI: magnetic resonance imaging

**Figure 6:**
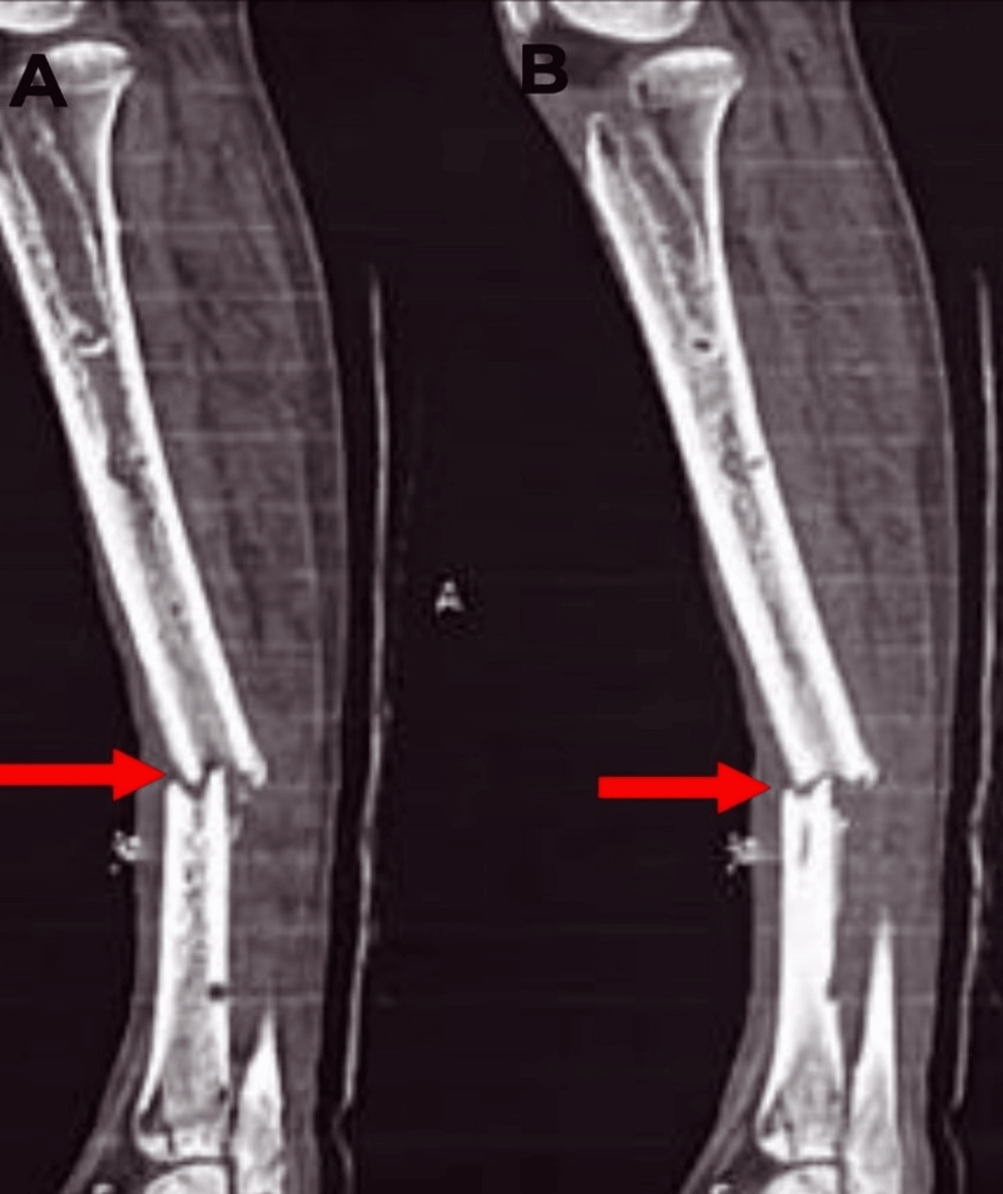
A-B) The sagittal section of the left leg CT scan. Red arrows show a complete, non-united displaced fracture in the middle one-third of the tibia shaft. CT: computed tomography

**Figure 7:**
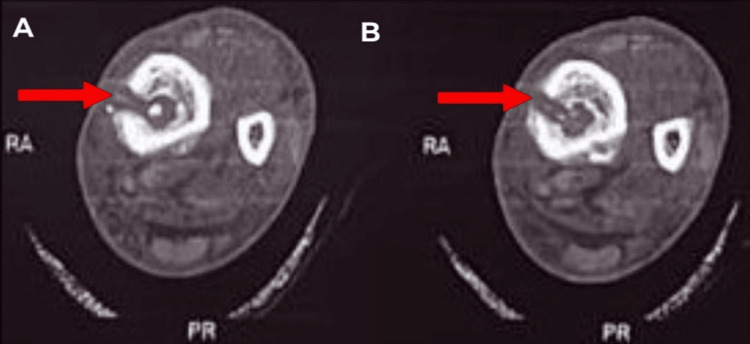
A-B) The axial section of the left leg CT scan. Red arrows show a cortical breach in the tibia shaft. CT: computed tomography

Left leg magnetic resonance imaging (MRI) and computed tomography (CT) scans were done to assess the extent of infection and confirm the non-union, respectively. The MRI showed a cortical breach in the lower one-third part of the tibia at two places through which fluid collection was seen (Figures 4-5). No sequestrum was seen. The CT scan showed complete, oblique, non-united displaced fractures in the middle one-third of the tibia and fibula, with medial displacement of the distal fractured fragments (Figures 6-7). The patient was diagnosed with a left middle one-third tibia and fibula-infected non-union.

**Figure 8:**
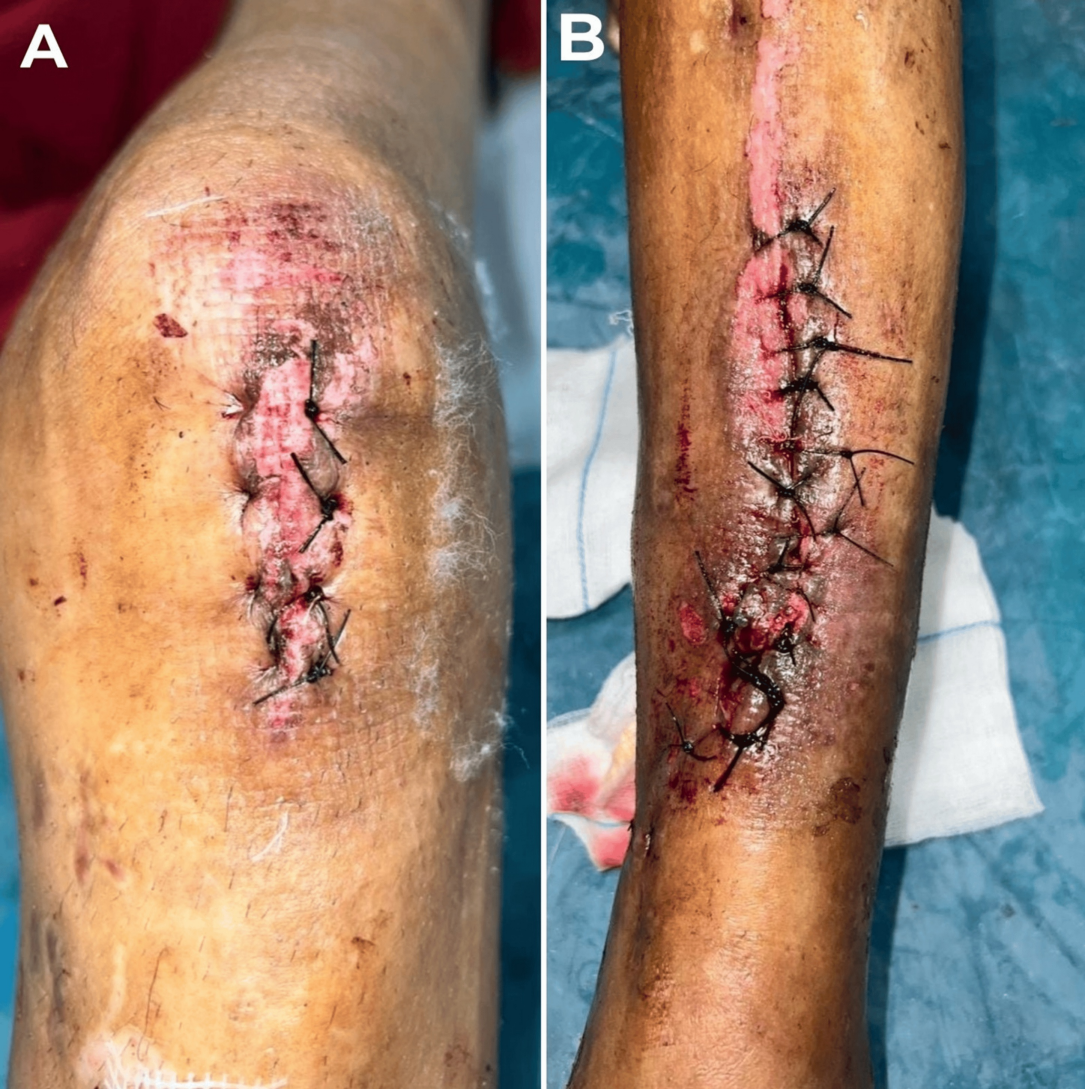
A-B) Clinical pictures of wounds post-debridement and antibiotic-impregnated cement-coated TENS nailing. TENS: Titanium Elastic Nail System

**Figure 9:**
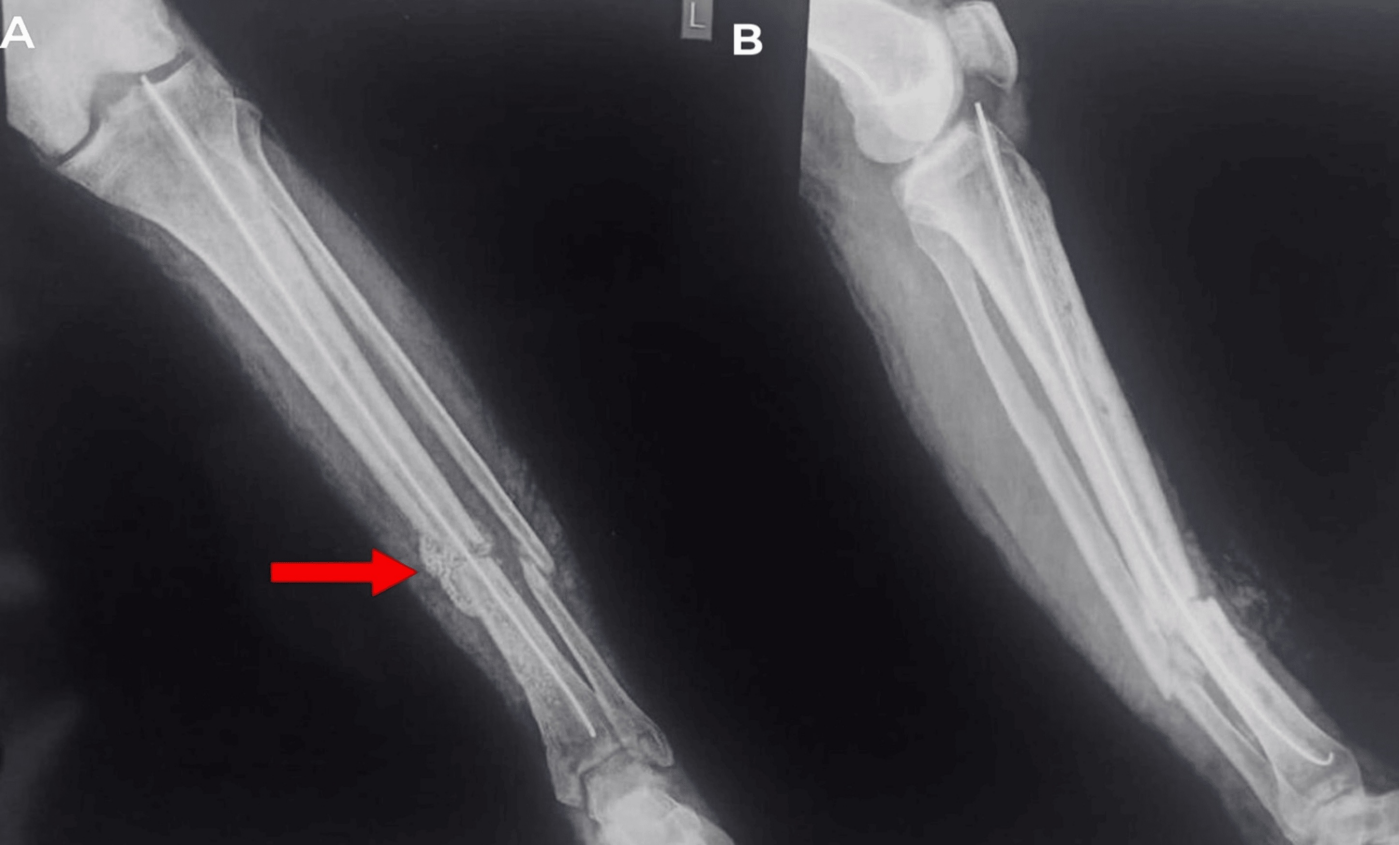
A-B) Postoperative X-rays of the left tibia and fibula AP and lateral view with antibiotic-impregnated cement-coated TENS insertion. A red arrow shows biodegradable calcium sulphate beads mixed with antibiotics. AP: anterior posterior; TENS: Titanium Elastic Nail System

Effective counselling of the patient and his relatives with regard to the cost and duration of the administration of IV antibiotics was done. Firstly, to control the infection, the patient was posted for debridement with antibiotic-impregnated cement-coated TENS (Titanium Elastic Nail System) insertion under spinal and epidural anaesthesia after routine lab investigations and the pre-anaesthesia checkup (Figure 8). The sinus tracts were excised, and the area was debrided. The fracture ends were freshened with a nibbler and a 2-cc syringe tube cut on either end. A 4.5-mm TENS nail was coated with bone cement mixed with vancomycin. An 8-mm-diameter nail was formed post-coating with the antibiotic-impregnated cement. The nail was inserted in the tibia post-reaming. Further, biodegradable calcium sulphate beads mixed with vancomycin were placed into the site of debridement. An intra-op sample was sent for culture sensitivity and histopathological examination (HPE). Closure was done, and the surgery was uneventful. A postoperative left tibia full-length AP and lateral X-ray were done (Figure 9). The patient was kept non-weight-bearing.

The intra-op sample culture report again showed MRSA positivity, which was sensitive to vancomycin, gentamicin, teicoplanin and linezolid. Postoperatively, intravenous vancomycin 1 g BD was administered for two weeks and linezolid 600 mg BD for three weeks. He was kept on oral linezolid for two weeks after consulting the infectious disease consultants. Weekly erythrocyte sedimentation rate (ESR), C-reactive protein (CRP), and white blood count (WBC) reports were analysed, which showed a declining trend. The suture removal was done at two weeks post-surgery, and the wound had healed with primary intention without any signs of dehiscence.

In this first stage of treatment to control the infection, which lasted for seven weeks, the patient underwent debridement, vancomycin-impregnated calcium sulphate beads insertion, and vancomycin-impregnated cement-coated TENS nail insertion. The patient was administered IV vancomycin for two weeks, IV linezolid for three weeks and oral linezolid for two weeks. The infection got under control by seven weeks, and the patient was kept non-weight-bearing for seven weeks.

**Figure 10:**
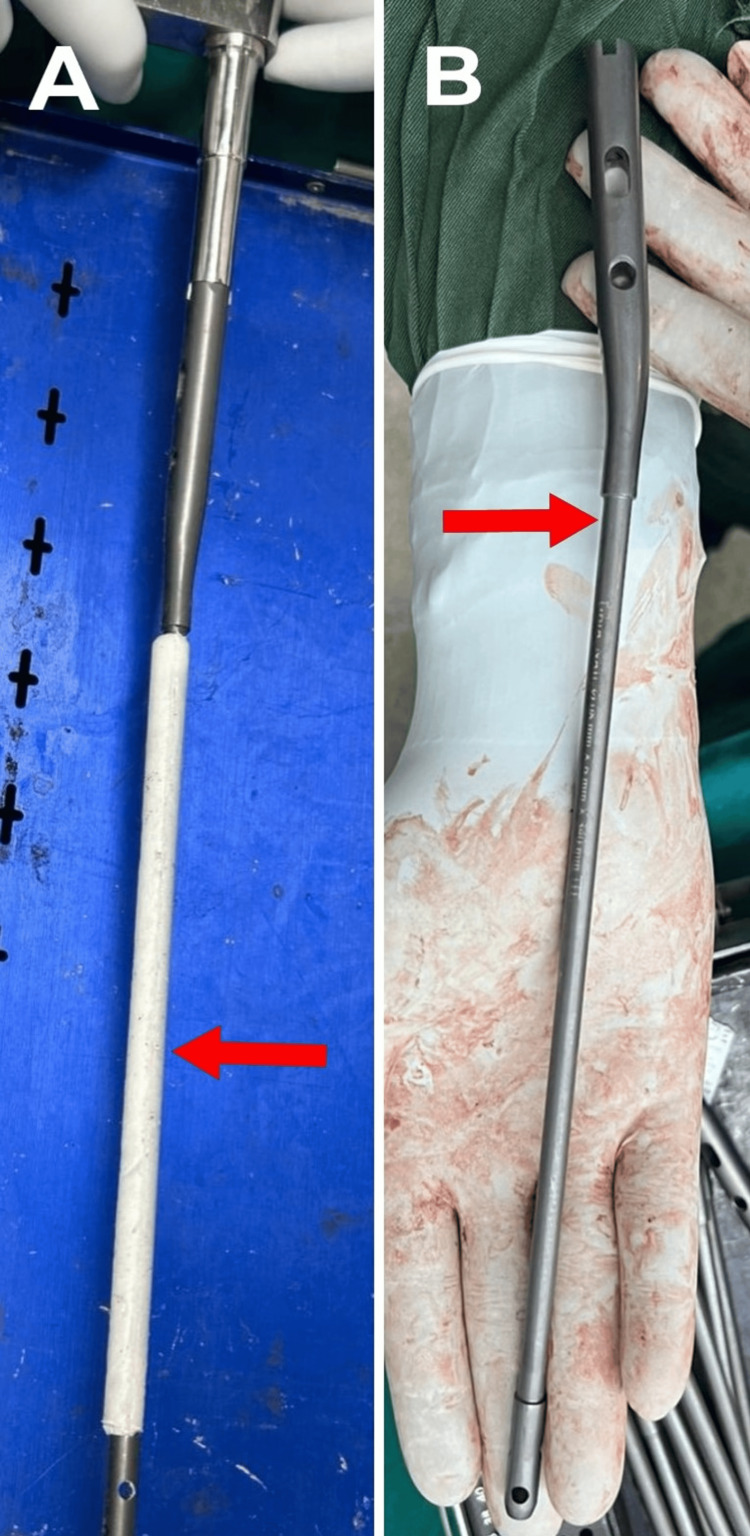
A) A red arrow shows antibiotic-impregnated cement coated over the solid intramedullary nail. B) A red arrow shows the specially made facet provided at the outer surface of the solid intramedullary nail.

As soon as the infection got under control (seen as four repeated samples of normal laboratory parameters done weekly) at seven weeks after the first surgery, the patient was further posted for TENS nail removal, re-debridement, and antibiotic-impregnated cement-coated solid intramedullary nailing. This solid IM nail had a specially made facet on its outside surface for cement coating (Figure 10B). The patient was taken up for surgery under spinal and epidural anaesthesia after routine lab investigations and pre-anaesthesia checkup. The antibiotic-impregnated cement-coated TENS nail was removed. A fibulectomy was done to maintain the length of the tibia. Sequential reaming was done with 8 mm and 9 mm solid reamers. An 8 mm × 34 mm solid slotted IM nail was coated with bone cement (incorporated into the specially made facet over its exterior surface) mixed with vancomycin (Figure 10A). An intra-op sample was collected and sent for culture sensitivity. The nail was inserted into the tibia. The sinus tract sites were reopened and debrided. Biodegradable calcium sulphate beads mixed with vancomycin were inserted into the debrided sinus tract sites, and the fibulectomy site and closure were done. The surgery was uneventful.

**Figure 11:**
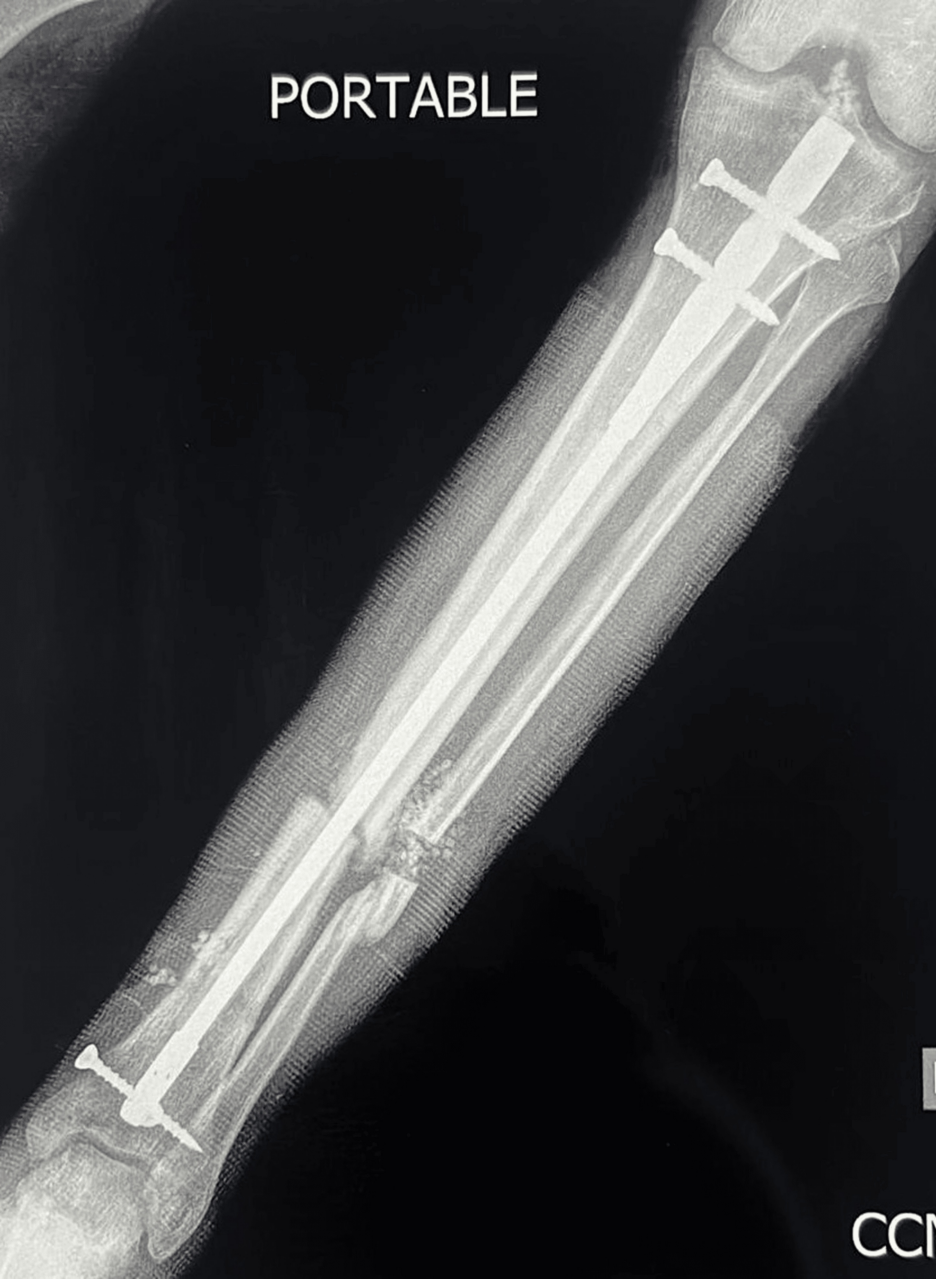
Postoperative X-ray of the left tibia and fibula AP view showing exchange nailing with a solid intramedullary nail with cement coating. AP: anterior posterior

**Figure 12:**
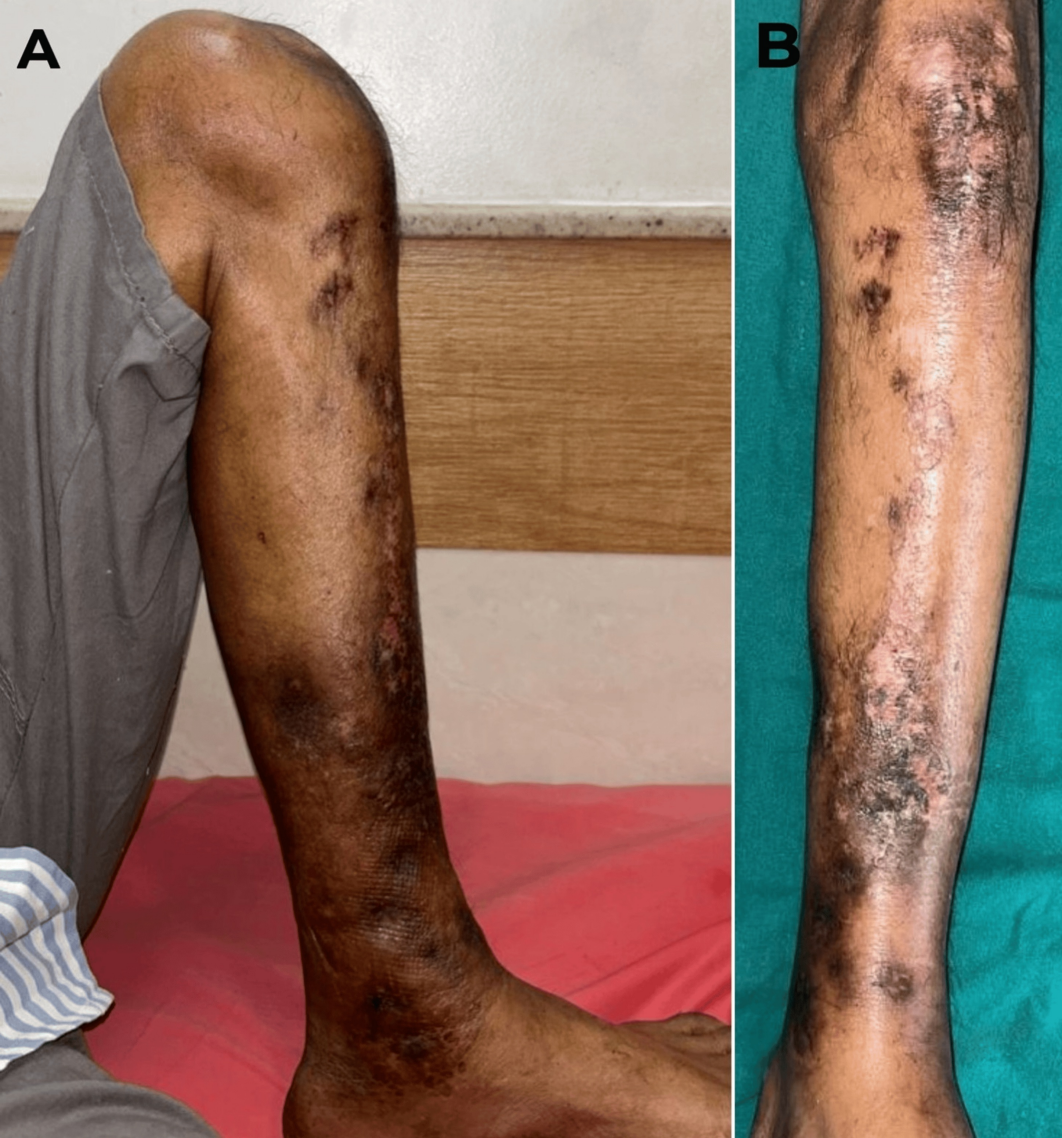
A) Satisfactory left knee flexion post-solid intramedullary nailing. B) A clinical picture of healed wounds from the surgery and the absence of sinuses.

A post-op left tibia full-length AP X-ray was done (Figure 11). The intra-op sample report showed no growth of any organism. IV teicoplanin 600 mg OD was administered for two weeks after consulting the infectious disease consultants. The suture removal was done two weeks post-surgery. There was no wound dehiscence. The patient was discharged on oral linezolid for one week. The patient had satisfactory knee flexion (Figure 12), and full weight-bearing was allowed.

In this second stage of treatment, which lasted for three weeks, to stabilise the bone and prevent a recurrence of infection, TENS nail removal, re-debridement, vancomycin-impregnated cement-coated solid IM nail insertion, and vancomycin-impregnated calcium sulphate beads insertion were done. The patient was administered IV teicoplanin for two weeks and oral linezolid for one week. The patient was allowed to bear full weight on his left lower limb.

**Figure 13:**
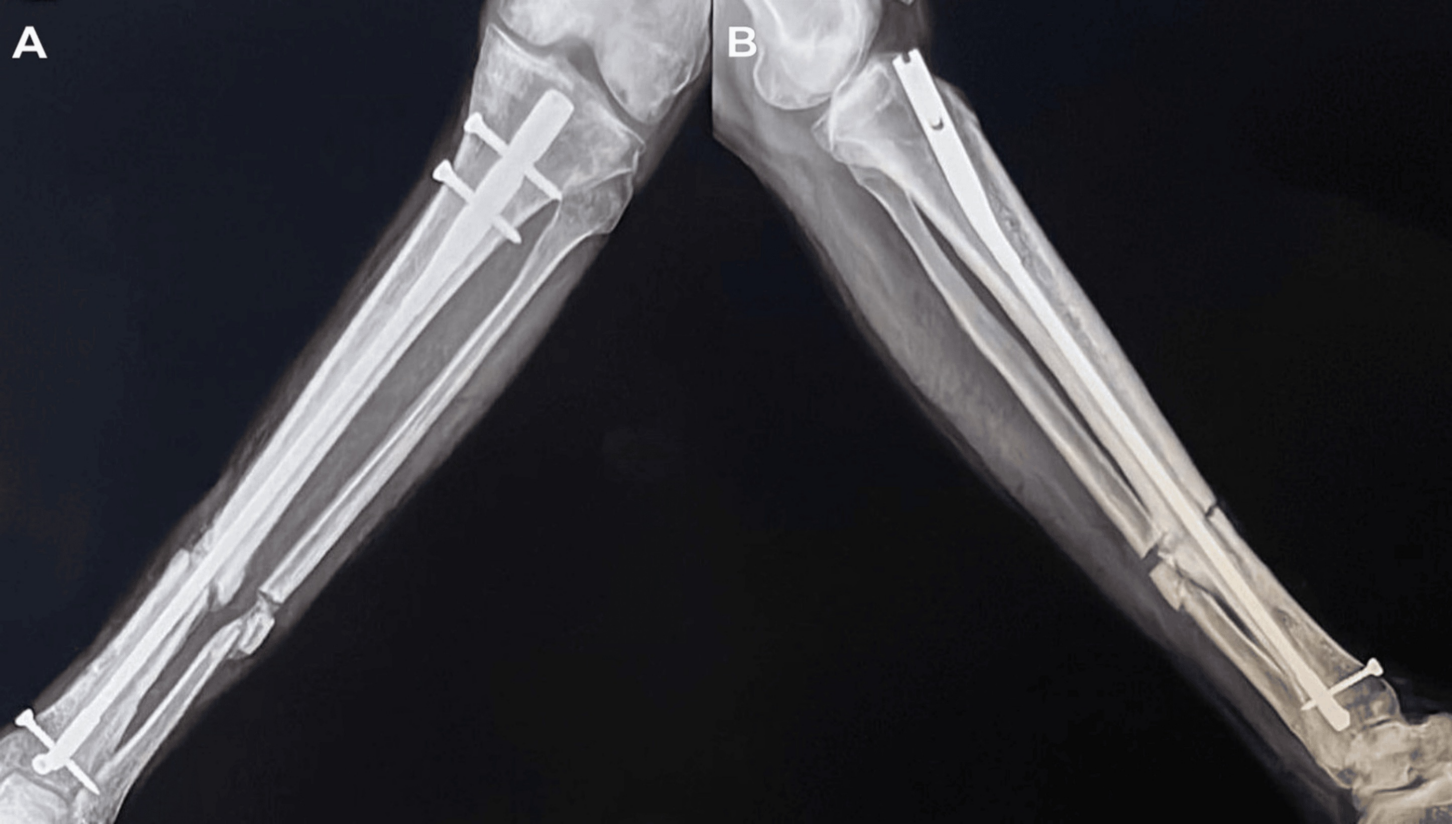
A-B) Left tibia and fibula AP and lateral X-ray views with signs of fracture union at two months post-solid intramedullary nailing. AP: anterior posterior

**Figure 14:**
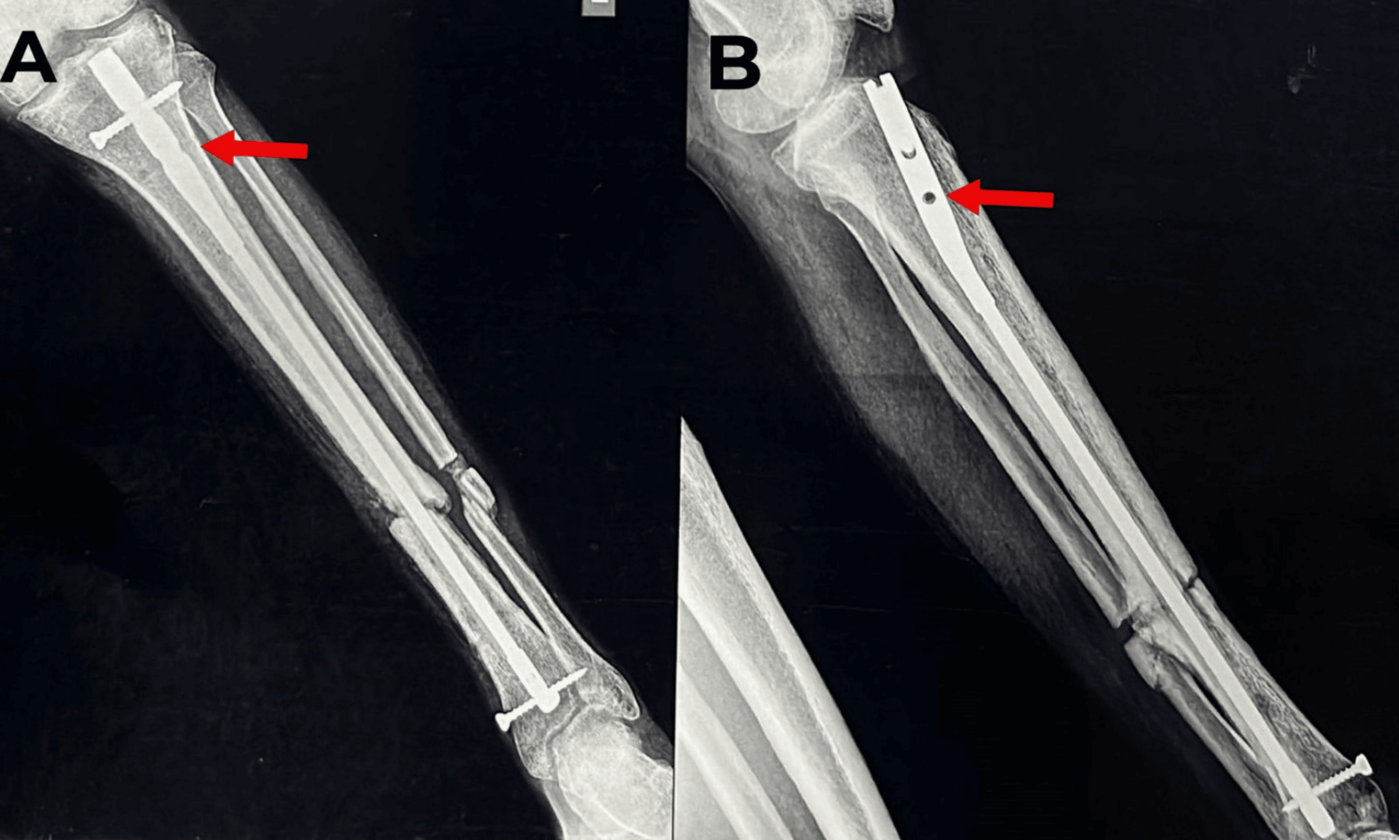
A-B) Left tibia and fibula AP and lateral X-ray views, respectively. Red arrows show dynamization. AP: anterior posterior

The patient came for follow-up every two weeks. ESR, CRP, and WBC were analysed every two weeks, which were within normal limits. The X-ray was repeated six weeks post-surgery, which showed signs of union (callous formation) (Figure 13). To further accelerate the union process, dynamization was done at eight weeks post-solid IM nailing (Figure 14).

**Figure 15:**
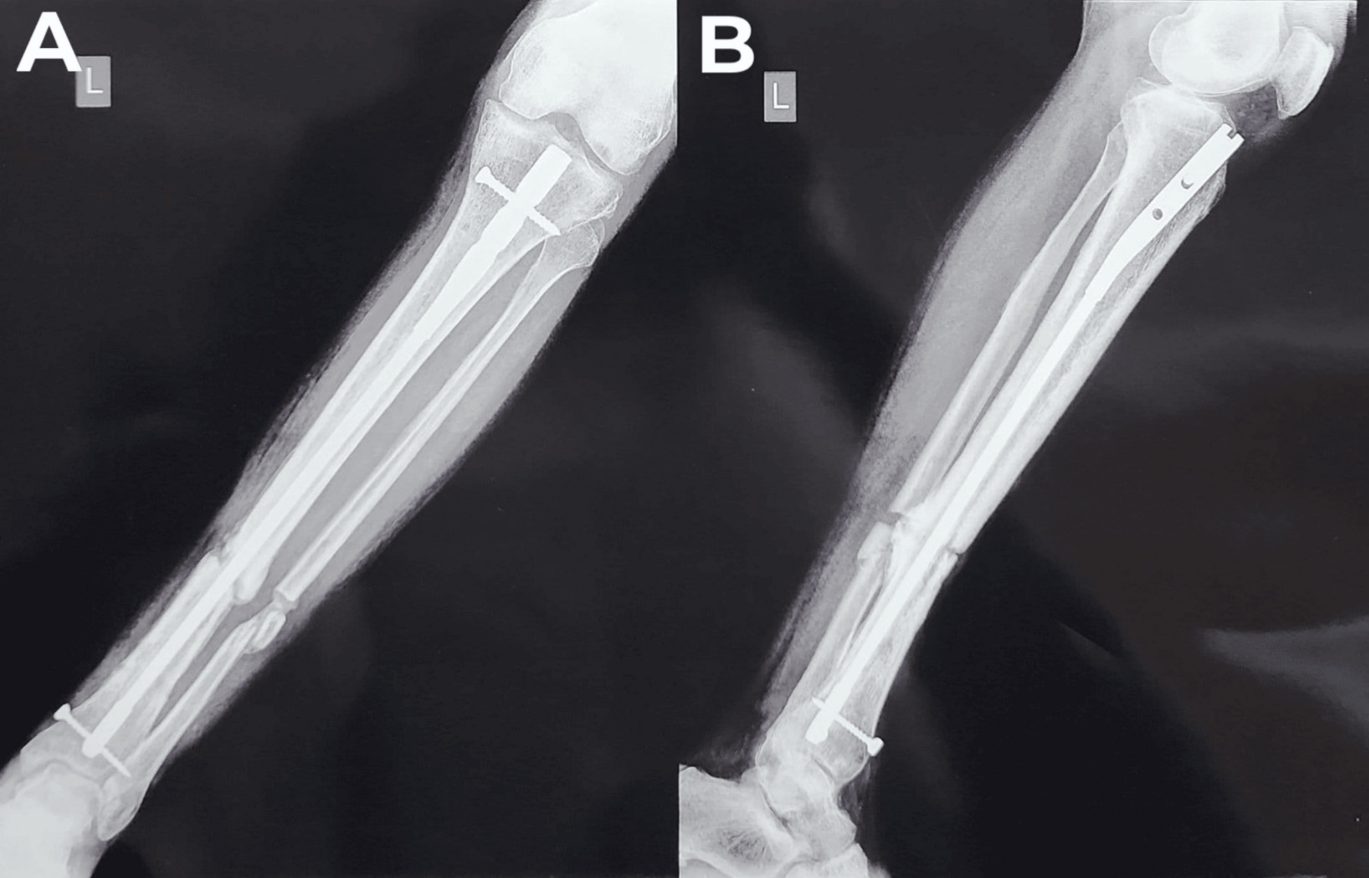
A) A left tibia and fibula AP X-ray with increased callus deposit at the non-union site. B) A left tibia and fibula lateral X-ray with increased callus deposit at the non-union site. AP: anterior posterior

**Figure 16:**
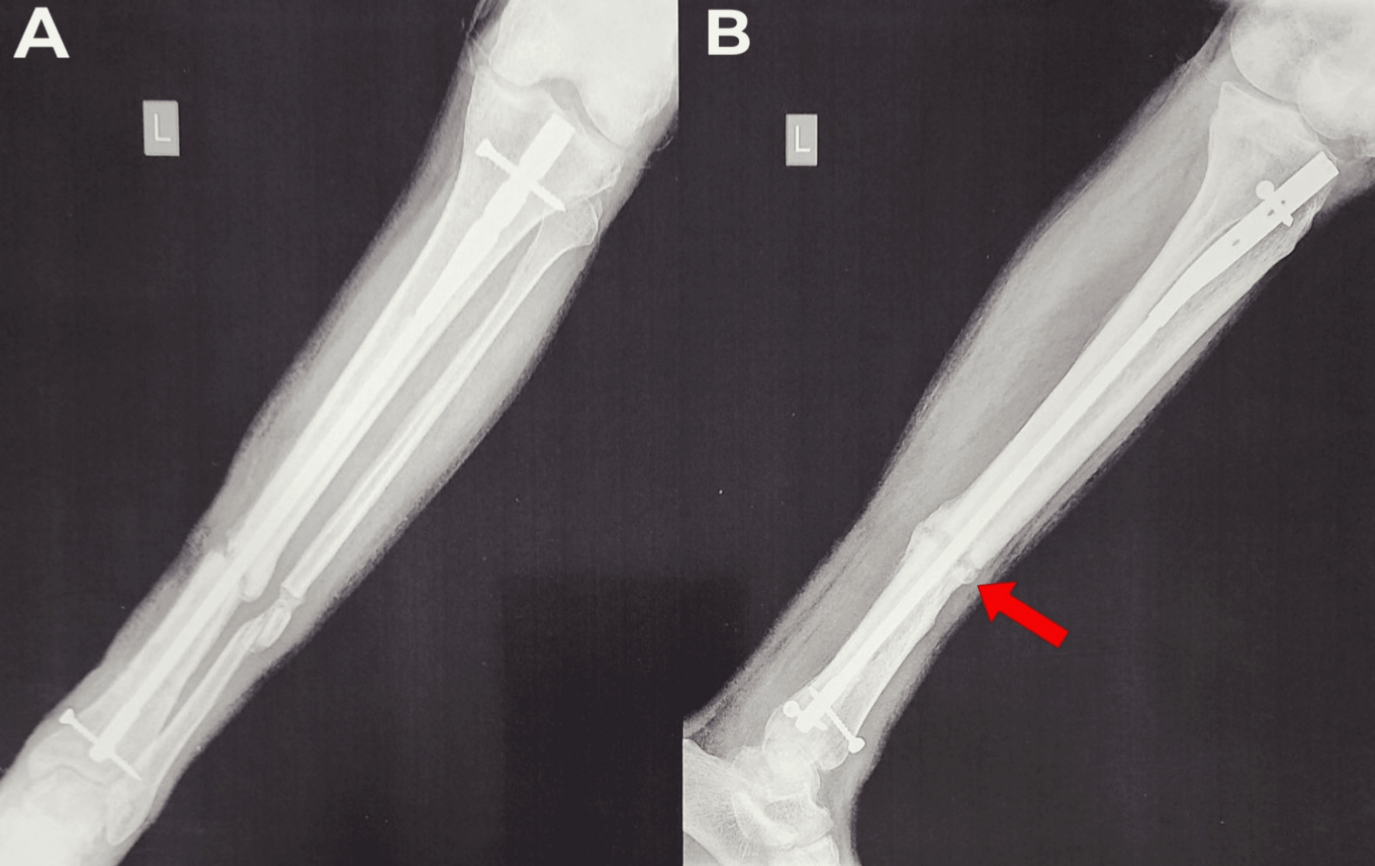
A) Left tibia and fibula full-length AP X-ray with good callus formation. B) Left tibia and fibula full-length lateral X-ray with increased callus deposit in anterior cortex region of the non-union site. A red arrow shows an increased callus deposit in the anterior cortex region of the non-union site. AP: anterior posterior

One-month post-dynamization, a repeat X-ray AP and lateral of the left tibia and fibula was done, which showed increased callus deposits at the non-union site (Figure 15). Another follow-up X-ray was taken at two months post-dynamization, i.e., five months post-antibiotic-impregnated cement-coated solid IM nailing, which shows good callus formation in the AP X-ray and increased callus deposit in the anterior cortex region of the non-union site, indicating progression of the bone union process (Figure 16). On clinical examination, there were no visible signs of infection, like discharging sinuses, redness, swelling, or a local rise in temperature. Also, the inflammatory markers, i.e., ESR, CRP, and total leukocyte count (TLC), were within the normal limits.

## Discussion

The cornerstones of treatment for infected non-union of the long bone are rigorous debridement, rigid fixation, and extended antibiotics. For the treatment of infected non-unions, numerous stepwise techniques have been developed. Intramedullary devices have shown promise in the secondary stage of bone healing as well as the main stage of infection management [8]. Infected non-union patients must be treated with measures to achieve stability and manage the infection before the bone can achieve union [9]. For the management of infected non-unions, there is currently no one standard therapeutic approach that is acknowledged worldwide. The two-step method of controlling the infection and then treating the non-union has been the standard for managing infected non-unions. Scarred muscle and subcutaneous tissue, as well as a thicker periosteum, envelop the infected foci within the bone. The bone is sclerotic and comparatively avascular. Systemic antibiotics are largely rendered useless by this avascular scar tissue sheath.

One recognised side effect of intramedullary nailing is intramedullary infection that results in non-union of the fracture [10]. The incidence of compound fractures is higher than that of closed fractures that are treated with intramedullary nailing [11]. Buchholz et al. were the ones who initially reported the use of antibiotic-impregnated cement [12]. The main benefit was reduced systemic side effects and a high local concentration of antibiotics [13]. Vancomycin has been the second most commonly utilised agent, behind gentamicin. When two antibiotics-gentamicin and vancomycin-are used with bone cement, their range of action is expanded, and their elution characteristics are improved [13]. When treating osteomyelitis, Klemm was the first to employ antibiotic cement beads [14]. Cement beads provide a high concentration of local antibiotics while simultaneously filling the dead space. The efficacy of cement beads loaded with antibiotics in managing bone infections is widely recognised. For intramedullary infections, cement beads have been employed. But after two weeks, they are challenging to remove and provide no mechanical stability [15]. In addition to offering all the benefits of cement beads, an antibiotic-impregnated cement-coated nail can also offer stability and ease of removal.

To manage infection, antibiotics must be delivered locally or systemically to the infection site. Excessive fibrosis is caused by prolonged infection and recurrent debridement, which reduces the permeability of antibiotics surrounding the non-union site [16]. Antibiotics administered locally therefore have significantly greater advantages than those administered systemically. In order to treat osteomyelitis and open fractures, it is well established that antibiotics can be administered locally using antibiotic-impregnated polymethylmethacrylate cement beads without causing systemic toxicity [16]. Nevertheless, because of the challenging removal posed by fibrous ingrowths, these antimicrobial cement beads cannot be inserted in the intramedullary canal and do not offer any support across the fracture site. A wide range of activity, heat stability, good cement elution qualities, and low allergenicity are desirable characteristics for antibiotics utilised for this purpose. Vancomycin plus gentamicin or tobramycin was once a common combination employed by researchers [5, 9]. Using two antibiotics at the same time contributes to the broader range of action.

Fixation, either internal or external, can provide stability across the fracture site. But there is evidence that external fixation is linked to a high frequency of pin-site infections, muscular contractures, and joint stiffness [17]. The Ilizarov fixator, while a great technique for treating infected non-union, requires a lot of assembly work, is technically complex, and is not well-liked by patients. Internally fixed implants can act as foreign bodies and potentially serve as a medium for infection through the creation of biofilms. The removal of infection with systemic antibiotics is hampered by the existence of biofilm and foreign bodies.

With the use of antibiotic-cement-coated nails, a two-stage non-union treatment process can be reduced to a single stage by delivering a high concentration of antibiotics at the non-union site while preserving stability there. Antibiotic cement nails also aid in preventing complications by allowing for early patient mobilisation and preventing pin-site infections, stiff joints, and muscle contractures. Many researchers have used this method to treat infected non-union patients with good results since Paley and Herzenberg’s 2002 initial report [18]. Paley and Herzenberg found that all nine instances in their small sample had infection control. In a 2007 study, Thonse and Conway reported that 85% of the patients in their large sample size of 52 had infection control [9]. The outcomes of several investigations varied with regard to bony union. Bony union was documented by Thonse and Conway in 73% of the patients where the index surgery was an antibiotic nail. Antibiotic cement rods were prepared by Thonse and Conway using interlocking nails. Fibrous growths and cement nail bonding might imprison the nail inside the medullary canal. Because live bacteria can theoretically remain on antibiotic-impregnated cement under in vitro conditions, prompt nail removal is advised once the infection has cleared up [19].

In an investigation conducted by Horn et al., solid nails exhibited a greater resistance to local infection than cannulated intramedullary nails, which exhibited characteristics akin to slotted nails [7]. Two key variables may have contributed to the nearly twofold increase in infection susceptibility observed in the hollow slotted nail compared to the solid nail in an animal experiment conducted by Melcher et al. The hollow area is an area that is either bloodless or poorly vascularised, where germs can thrive in the presence of debris and necrotic tissue and where the host’s defensive mechanisms have restricted access. Additionally, the hollow nail's implant surface is about twice as large as the solid nail's, which increases the possibility of bacterial adherence [20].

To summarise this discussion, we would like to say that controlling the infection should be the top priority in order to treat any infected non-union and then stabilise the fracture site to promote bone union. To achieve this, in the first stage of treatment, we debrided the sinus tracts, introduced antibiotic-impregnated calcium sulphate beads to control the infection in the soft tissues, and placed antibiotic-impregnated TENS nail in the tibia to control the infection in the medullary canal of the tibia. This made sure that there was a sustained release of local antibiotics through the calcium sulphate beads and the cement. In a study by Ryuji Mori et al., it was seen that the antibiotics were released locally for up to eight weeks post-implantation of such antibiotics impregnated with calcium sulphate beads and cement [21]. This also ensured that there were fewer chances of the development of antibiotic resistance by reducing prolonged systemic antibiotic administration and delivering local antibiotics. Repeated analysis of infective markers like WBC, ESR, and CRP was done to check whether the infection was under control. And in the second stage of our treatment, we removed the TENS nail, redebrided the sinus tract sites, and then stabilised the fracture site with antibiotic-impregnated cement-coated solid IM nail. Coating of the nail in this stage too was done to prevent any biofilm formation and prevent the recurrence of infection. Further, the use of specially designed solid IM nail with facet for cement coating helped in maintaining uniform diameter of the nail throughout the medullary canal of the tibia, thus providing good stabilisation with superior infection controlling modality than any other IM nail. Also, germ sensitivity and MIC should be taken into account before choosing the antibiotic regime and dosage to treat the infection. However, to fully comprehend its efficacy in reducing infection and fostering bone union, we will need to continue longer follow-up and study a larger sample size.

## Conclusions

An easy, affordable, and successful treatment for infected non-union of the tibia is antibiotic cement impregnated nailing. It has strong patient compliance and removes the problems associated with external fixators, which makes it superior to them. A few benefits of this approach are early weight-bearing, stabilisation of the fracture, local antibiotic treatment, and the potential for accelerated rehabilitation. Additionally, by lowering the requirement for continuous antibiotic medication, it may lessen the chance that antibiotic resistance may arise. Because this procedure is less technically demanding, it can be implemented at any general orthopaedic facility. In this study, an intramedullary nail that has a specially made facet at its exterior surface for coating with antibiotic-impregnated cement was used. Thus, it appears that this solid intramedullary nail custom-made for antibiotic-impregnated cement coating is an excellent mode of local antibiotic treatment and stabilisation of the fracture. To fully comprehend its efficacy in reducing infection and fostering bone union, we will need to continue longer follow-up and study a larger sample size.
